# Integrating CRISPR functional genomics with admixture-aware psychotropic pharmacogenetics in Brazil

**DOI:** 10.3389/fphar.2026.1730641

**Published:** 2026-06-04

**Authors:** Brenda Bassan Rodrigues, Jasmine Coura Bittencourt, Vitória Gorga Machado dos Santos, Carlos André da Veiga Lima-Rosa Costamilan, Marcos Edgar Herkenhoff

**Affiliations:** 1 Laboratory of Molecular Genetics, Center for Higher Education of the Southern Region (CERES), Santa Catarina State University (UDESC), Santa Catarina, Laguna, Brazil; 2 Herkenhoff Molecular Consulting, São Paulo, Brazil

**Keywords:** antidepressants, antipsychotics, CRISPR/Cas9, personalized psychiatry, pharmacogenetics

## Abstract

Variability in the clinical response to psychotropic drugs remains a major barrier to effective psychiatric care. Pharmacogenetics offers a powerful framework for individualizing antidepressant and antipsychotic therapy, yet its implementation is complicated in genetically diverse populations. In Brazil, centuries of admixture among European, African, and Indigenous peoples have created a highly heterogeneous genomic landscape that limits the direct applicability of international pharmacogenetic guidelines. This review synthesizes evidence on ancestry-dependent allele frequencies, regional genetic gradients, and their clinical implications for psychotropic prescribing. It further discusses how integrating CRISPR functional genomics with admixture-aware psychiatry can experimentally validate population-specific variants, refine metabolizer phenotype classification, and support the development of ancestry-informed therapeutic guidelines. By combining genome editing, pharmacogenomic profiling, and regional admixture data, this integrative approach can improve dose prediction, reduce adverse drug reactions, and promote health equity. Ultimately, the convergence of pharmacogenetics, functional genomics, and precision medicine initiatives positions Brazil as a global model for equitable, ancestry-aware psychopharmacology.

## 1 Introduction

Interindividual variability in the response to psychotropic medications remains one of the central challenges in contemporary psychiatry. Although pharmacogenetics has emerged as a key strategy to explain differences in drug metabolism, efficacy, and tolerability, most of the clinically actionable evidence currently focuses on a limited number of variants in genes such as CYP2D6 and CYP2C19 (Chenchula et al., 2025). These variants influence the pharmacokinetic profiles of antidepressants and antipsychotics and have motivated the development of international guidelines for genotype-informed prescribing. However, the clinical utility of these guidelines is constrained by important gaps: the lack of functional validation for many rare or population-specific alleles, limited predictive accuracy for complex pharmacodynamic responses, and the underrepresentation of admixed populations in genomic databases (Baldacci et al., 2023; Zhang and Yue, 2025).

Pharmacogenetics is a scientific field that investigates how individual genetic variations influence drug response, constituting one of the pillars of personalized medicine. In psychiatry, its application is particularly relevant due to the considerable interindividual variability in the efficacy and safety of psychotropic drugs, such as antidepressants and antipsychotics (Radosavljevic et al., 2023). Major depressive disorder (MDD) demonstrates substantial treatment heterogeneity: only about 30% of patients achieve remission after the first antidepressant trial, while 50%–60% exhibit inadequate response, often requiring multiple therapeutic adjustments (Arnone et al., 2022; Ramaraj et al., 2023; Tesfamicael et al., 2023; Wang et al., 2023).

Beyond inherited genetic variation, phenoconversion—a phenomenon where genotypic normal metabolizers (NMs) are converted into functional poor metabolizers (PMs) via strong CYP2D6 inhibitors (e.g., fluoxetine, paroxetine, bupropion)—further complicates phenotype prediction. This drug-induced suppression of enzymatic activity elevates plasma concentrations of co-administered substrates (e.g., tricyclic antidepressants, aripiprazole), increasing toxicity risk independent of genotype (De Brabander et al., 2024; Bousman et al., 2023a). This phenomenon, observed in more than 30% of Brazilian psychiatric patients treated with potent CYP2D6 inhibitors (De Brabander et al., 2024; Patel et al., 2024), demands integrated approaches that combine pharmacogenetic testing with systematic drug–drug interaction screening.

This variability can be partly explained by polymorphisms in genes encoding drug-metabolizing enzymes, such as CYP2D6 and CYP2C19, as well as genes for neurotransmitter transporters and receptors, which influence the pharmacokinetics and pharmacodynamics of these medications. These factors modulate plasma drug concentrations and, consequently, clinical efficacy and the risk of adverse effects (van Westrhenen et al., 2020). The clinical relevance of genetic polymorphisms in CYP2C19 and CYP2D6—key enzymes in the metabolism of selective serotonin reuptake inhibitors (SSRIs), serotonin-norepinephrine reuptake inhibitors (SNRIs), and tricyclic antidepressants—has been well documented.

Genotype-based metabolizer phenotypes (poor, intermediate, extensive, ultra-rapid) can significantly alter antidepressant plasma concentrations, affecting both therapeutic effectiveness and side-effect risk (Li et al., 2024). Additional elements, including environmental conditions, comorbidities, and epigenetic mechanisms, also contribute to this complexity. In the United States, adverse drug reactions are estimated to account for over 100,000 deaths and more than two million hospitalizations annually. Meta-analyses and randomized controlled trials (RCTs) indicate that pharmacogenomic-guided treatment yields modest but statistically significant improvements: approximately 18% higher response rates and 37% higher remission rates at 8 weeks versus treatment as usual (Bunka et al., 2023; Milosavljević et al., 2024; Tesfamicael et al., 2023).

A critical aspect in this context is the classification of patients as ultra-rapid or poor metabolizers, whose genetic profiles significantly alter the absorption and metabolism of psychotropic drugs, thereby requiring individualized clinical approaches (Radosavljevic et al., 2023; Zhao et al., 2021). Although pharmacogenetics has enabled remarkable advances, it remains insufficient to fully explain the wide range of clinical responses observed in mental disorders (Bertollo et al., 2025; Butler, 2018; Principi et al., 2023).

In this context, CRISPR/Cas9 (Clustered Regularly Interspaced Short Palindromic Repeats associated protein 9) technology has emerged as a versatile experimental platform capable of functionally interrogating pharmacogenetic variants and refining the mechanistic understanding of psychotropic drug response (Aderinto et al., 2026; Khan et al., 2025). Rather than being framed primarily as a therapeutic tool, CRISPR enables the precise introduction, deletion, or modulation of alleles of interest within isogenic cellular models (Khan et al., 2025). These engineered systems make it possible to directly assess how specific variants influence enzymatic activity, transporter kinetics, receptor signaling, or regulatory dynamics relevant to antidepressant and antipsychotic action—an approach that is particularly valuable for variants of uncertain significance and for haplotypes underrepresented in European-ancestry cohorts, which continue to dominate current pharmacogenomic guidelines (Blambila et al., 2026; Lorvellec et al., 2024; Vasiliu, 2023).

Building on these methodological advances, gene-editing strategies have widened the scope of functional investigation by enabling targeted perturbation not only of coding regions but also of regulatory elements such as lncRNAs and miRNAs (Akinci et al., 2021; Li et al., 2025). These non-coding components play critical roles in neuropsychiatric pathophysiology, including schizophrenia and bipolar disorder, and their manipulation provides a route to disentangle how upstream regulatory architecture shapes pharmacological response (Martin et al., 2019). The precision, efficiency, and adaptability of CRISPR/Cas9 across cellular and organismal models, therefore position gene editing as a promising framework for generating the functional evidence required to bridge existing gaps in pharmacogenetics and to advance more inclusive, mechanism-grounded models of psychotropic drug variability (Ji et al., 2026; Khan et al., 2025).

The need for functional validation is especially pronounced in highly admixed populations, including those of Latin America. In Brazil, the extensive mixture of European, African, and Indigenous ancestries generates unique combinations of pharmacogenetic alleles and copy-number variants that challenge phenotype prediction based solely on foreign guidelines (Suarez-Kurtz, 2010; Suarez-Kurtz et al., 2012). This genomic context complicates metabolic phenotype assignment, alters allele frequency expectations, and introduces uncertainty into standard genotype-to-phenotype algorithms such as those used by CPIC and DPWG. CRISPR-based functional genomics provides an opportunity to test the real biological effects of these variants in controlled models, thereby producing evidence better aligned with the genetic architecture of admixed populations (Lee et al., 2025).

This review synthesizes current knowledge at the intersection of psychotropic pharmacogenetics, CRISPR/Cas9 functional genomics, and population diversity. It examines how CRISPR methodologies—knock-in/knock-out editing, CRISPR interference and activation (CRISPRi/a), pooled screens, and iPSC-derived neuronal models—can elucidate the functional impact of pharmacokinetic and pharmacodynamic variants. It also discusses the implications of genetic admixture for clinical implementation, emphasizing the need to incorporate population-specific data into precision psychiatry frameworks. By integrating these dimensions, the review highlights how CRISPR-enabled functional evidence can enhance the accuracy, equity, and translational value of pharmacogenetic strategies in psychiatry, particularly in settings characterized by high genomic heterogeneity.

To our knowledge, no previous review has systematically integrated (i) admixture-dependent pharmacogenetic variation in Brazil, (ii) CRISPR-based functional genomics of psychotropic drug response, and (iii) the translation of such evidence into ancestry-aware prescribing frameworks. Here, we propose a conceptual workflow that links variant discovery in admixed Brazilian cohorts to CRISPR-enabled functional validation and, ultimately, to locally calibrated clinical guidelines. This perspective explicitly reframes CRISPR/Cas9 not as a therapeutic tool, but as an experimental engine for mechanistic validation in the context of Brazil’s unique genomic diversity.

## 2 Pharmacogenetics of psychotropic drugs: Current landscape

Psychotropic drug response arises from variation in both pharmacokinetic and pharmacodynamic pathways. Among pharmacokinetic determinants, polymorphisms in CYP2D6 and CYP2C19 remain the most clinically actionable, influencing the metabolism of SSRIs, SNRIs, tricyclic antidepressants, and several antipsychotics (Austin-Zimmerman et al., 2021; Blambila et al., 2026; Mangalagiu et al., 2026). These variants define metabolizer categories—poor, intermediate, normal/extensive, or ultra-rapid—which modify plasma concentrations and shape efficacy and tolerability (Serretti et al., 2026). Innovation in psychotropic drug development has slowed in recent decades, reinforcing the value of pharmacogenetics as a strategy to tailor treatment according to interindividual genomic variability (Radosavljevic et al., 2023).

Pharmacodynamic variation also contributes substantially to treatment heterogeneity. Genes such as SLC6A4, HTR2A, DRD2, COMT, and BDNF have been associated with differential responses to antidepressants and antipsychotics, although replication across populations remains inconsistent, illustrating the limitations of single-gene associations and the influence of environmental, epigenetic, and polygenic factors (Becker and Funk, 2018; Illi et al., 2009; Zhao et al., 2019; Dattani et al., 2022; Taylor et al., 2023). Meta-analyses of SLC6A4 indicate that carriers of the long (L) allele may experience improved SSRI outcomes, yet large multicenter studies such as STAR*D have not uniformly confirmed these findings, underscoring heterogeneity in serotonergic regulation (Sreeja et al., 2024; Stein et al., 2021; Kraft et al., 2007).

Structural and rare variants further complicate pharmacogenetic interpretation. CYP2D6 copy-number variation, for example, affects metabolic phenotype but is often missed by standard assays. Phenoconversion—frequently induced by CYP2D6-inhibiting drugs such as fluoxetine or paroxetine—can reclassify genotypic normal metabolizers as functional poor metabolizers, impacting more than 40% of patients in some cohorts (de Brabander et al., 2024; Hanna et al., 2025; Patel et al., 2024; Zhu et al., 2017).

**FIGURE 1:**
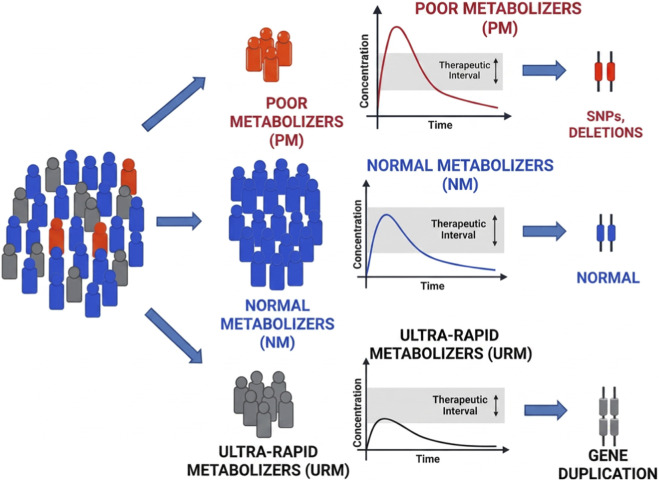
Representation of three distinct metabolic profiles—Ultra-rapid metabolizer (URM), Normal metabolizer (NM), Poor metabolizer (PM) for CYP2D6 (for CYP2C19 there are five phenotypes: poor, intermediate, normal/extensive, rapid and very rapid) — in response to the same psychotropic drug. Individuals with different genetic profiles exhibit variations in plasma drug concentrations, affecting efficacy and the incidence of adverse effects (Source: Adapted from Hicks et al., 2017; PharmGKB, 2025).

These complexities are reflected in international recommendations such as those from CPIC and DPWG, which provide genotype-based dosing guidance for medications metabolized by CYP2D6 and CYP2C19. Ultra-rapid metabolizers may clear drugs like escitalopram too quickly, reducing efficacy, while poor metabolizers show elevated plasma levels and greater adverse-effect risk (Figure 1) (de Leon et al., 2006; Kee et al., 2023; van Westrhenen et al., 2020; Bousman et al., 2023a; Shubbar et al., 2024; Bousman et al., 2023a; Hicks et al., 2017). These metabolizer categories, along with the population-level frequencies of key pharmacogenetic alleles in European, African, Indigenous, and admixed Brazilian groups, are summarized in Table 1, which complements the metabolic profiles illustrated in Figure 1. The allele frequencies presented in Table 1 are approximate, derived from heterogeneous sources, and are intended for conceptual illustration of admixture patterns rather than for use in individual-level clinical decisions or precise epidemiological modeling.

**TABLE 1:** Admixture-dependent allele frequencies of key pharmacogenetic variants in Brazil, compared with major ancestral groups.

Gene	Variant	Functional effect	European ancestry frequency (%)^1^	African ancestry frequency (%)^1^	Indigenous amerindian ancestry frequency (%)^1^	Estimated weighted brazilian frequency (%)^2^	Key references^3^
CYP2D6	*4(non-functional)*	Loss-of-function; PM	8–10	12–15	5–8	10–12	Bonifaz-Peña et al. (2014), Suarez-Kurtz et al. (2012), Suarez-Kurtz et al. (2014)
CYP2D6	Gene duplications	Ultra-rapid metabolism	3–5	1–3	2–4	3–5	Suarez-Kurtz, (2010), Bonifaz-Peña et al. (2014)
CYP2C19	*2(reduced function)*	Decreased metabolism	28–32	15–20	10–15	22–28	Suarez-Kurtz et al. (2012), Fernandes et al. (2021)
CYP2C19	*17(increased function)*	Rapid/ultra-rapid metabolism	25–30	10–15	8–12	18–24	Suarez-Kurtz, (2010), Bonifaz-Peña et al. (2014)
CYP3A5	*3 (loss of function)*	PM phenotype for CYP3A5	85–95	30–40	60–70	55–75	Bonifaz-Peña et al. (2014); Suarez-Kurtz et al. (2014)
SLC6A4	5-HTTLPR S allele	Reduced transporter expression	42–48	35–42	30–38	40–45	Crawford et al. (2013), Bonifaz-Peña et al. (2014)
DRD2	Taq1A	Altered receptor density	30–40	20–30	15–25	25–35	Fernandes et al., 2021
COMT	Val158Met	Altered dopamine degradation	25–32	18–25	20–30	20–28	Taheri et al., 2023; Suarez-Kurtz et al. (2012)
NAT2^4^	5 (slow acetylator)	Reduced acetylation capacity → slow acetylator	40–48	28–35	22–28	35–45	Suarez-Kurtz et al. (2014) Fernandes et al. (2021)
SLCO1B1^4^	5 (c.521T>C, reduced function)	Decreased hepatic uptake of statins and other drugs	12–16	6–10	4–8	10–14	Suarez-Kurtz et al. (2014) Ahmad et al. (2024)

1 Ancestral frequencies represent approximate ranges from reference panels for Iberian European, West-African, and Native American/Indigenous groups commonly used in Brazilian admixture studies (e.g., 1000 Genomes, local reference panels).

2 Brazilian weighted frequencies are rough estimates based on average national admixture proportions (∼51% European, 43% African, 6% Indigenous) and published regional datasets; values are intended for illustration of admixture impact rather than for clinical decision-making.

3 Key references are illustrative and should be aligned with the specific datasets used in the manuscript (e.g., refargen, regional cohort studies).

4 NAT2 and SLCO1B1 are suggested additions to broaden coverage of clinically relevant pharmacokinetic genes commonly co-prescribed with psychotropics (e.g., isoniazid, methotrexate, statins).

Advances in sequencing technologies offer opportunities to capture rare alleles and structural variants with higher precision, while emerging strategies such as CRISPR-Cas9 enable functional interrogation and potential correction of pathogenic variants that affect drug metabolism (Azeez et al., 2024; Kim et al., 2024; Satam et al., 2023). Integration of genomic, epigenomic, and microbiome data is expected to further refine personalized prescribing, although implementation requires clinician training, regulatory frameworks, and incorporation into national health systems (Molla and Bitew, 2024).

Recent work in psychiatric genetics emphasizes that reliance on CYP2D6 and CYP2C19 alone is insufficient for clinical decision-making. (Gutiérrez-Rodríguez et al., 2023) argue for polygenic frameworks capable of capturing the broader architecture influencing psychotropic response. Polygenic algorithms synthesize information across multiple genes to improve predictive accuracy and treatment selection, reducing the reliance on trial-and-error prescribing and potentially improving patient outcomes (Gutiérrez-Rodríguez et al., 2023).

Allele frequency ranges were compiled from multiple Brazilian and Latin American cohorts with varying sampling strategies and genotyping platforms (see key references in Table 1). For clarity and due to space constraints, we do not provide cohort-specific estimates or confidence intervals; future work should establish standardized, region-specific frequency maps based on harmonized methodologies. Brazilian weighted frequencies were approximated using average national admixture proportions (∼51% European, 43% African, 6% Indigenous) applied to ancestral frequency ranges; they should be interpreted as coarse, didactic approximations.

## 3 CRISPR/Cas as a functional genomics platform for pharmacogenetics

CRISPR/Cas9 (Clustered Regularly Interspaced Short Palindromic Repeats) is a versatile gene-editing platform that enables precise genomic modification, including targeted nucleotide substitutions, controlled gene disruption, and programmable modulation of regulatory elements (Doudna and Charpentier, 2014; Jinek et al., 2012). Within psychiatric pharmacogenetics, its primary value lies in providing experimental systems capable of testing how specific genetic variants influence metabolism, transport, or cellular response to psychotropic medications. Variants in *CYP2D6*, *CYP2C19*, *SLC6A4*, and *HTR2A*—which remain central to antidepressant and antipsychotic pharmacokinetics and pharmacodynamics—can be introduced into isogenic cell lines to characterize their mechanistic effects (Alchakee et al., 2022).

Rather than serving as a therapeutic tool, CRISPR contributes to pharmacogenetics by enabling the functional validation of alleles whose clinical impact cannot be inferred from association studies alone (Rusciano, 2026; Srivastav et al., 2025). Knock-in models allow researchers to introduce metabolizer variants, copy-number changes, or population-specific alleles and directly quantify resulting changes in enzyme activity, transporter function, or receptor signaling. When edited lines display altered sensitivity, accumulation, or downstream transcriptional responses to psychotropic drugs, these data help clarify whether such variants meaningfully affect treatment outcomes (Dominguez et al., 2016). This mechanistic insight is particularly relevant for variants that are rare globally but frequent in admixed populations, where predictive algorithms based on European-ancestry cohorts may fail.

CRISPR-based approaches have also expanded the capacity to model pharmacodynamic mechanisms. Studies such as (Kurishev et al., 2022) demonstrate how editing tools can dissect the contribution of psychiatric-risk genes to neuronal physiology. Although these investigations primarily address disease biology rather than drug metabolism, their methodological advances—especially the use of isogenic induced pluripotent stem cell (iPSC)-derived neurons—are directly applicable to pharmacogenetics. Editing risk variants or regulatory elements in serotonergic or dopaminergic neurons enables controlled testing of how specific alleles influence cellular responses to SSRIs, SNRIs, or antipsychotics, thereby supporting causal inference beyond traditional genome-wide association studies (Shi et al., 2012).

CRISPR interference (CRISPRi) and activation (CRISPRa) further expand these possibilities by modulating gene expression without altering the underlying DNA sequence (Gilbert et al., 2014). These approaches are particularly suited to evaluating regulatory variants that influence expression levels of pharmacogenes, such as promoter and enhancer polymorphisms in *SLC6A4*, *COMT*, or *HTR2A*. High-throughput CRISPRi/a screens in iPSC-derived neurons allow the simultaneous interrogation of dozens to hundreds of loci, enabling systematic identification of genetic modifiers that shape synaptic activity, intracellular signaling, and psychotropic drug response (Tian et al., 2019). When combined with high-content phenotyping—RNA sequencing, proteomics, morphology, electrophysiology—these screens generate integrated maps of the pathways mediating therapeutic effects or adverse outcomes (Norman et al., 2019).

Such polygenic screening strategies are especially relevant for psychopharmacology, where drug response emerges from multi-gene networks rather than single variants. By exposing CRISPR-perturbed neuronal libraries to SSRIs or antipsychotics, researchers can identify gene–drug interactions, characterize resistance mechanisms, and prioritize candidate variants for clinical investigation (Tian et al., 2019). These functional datasets complement GWAS findings and help to refine which loci exert biologically meaningful effects on pharmacodynamics. Table 2 summarizes key CRISPR/Cas9 functional genomics studies that have experimentally characterized pharmacogenetic variants or gene networks relevant to psychotropic drug response. These studies demonstrate how genome editing, CRISPRi/a modulation, and pooled screening platforms enable causal inference and mechanistic validation of variants identified through GWAS or clinical association studies. Such approaches are particularly valuable for testing population-specific or rare alleles, including those enriched in admixed populations such as Brazil.

**TABLE 2:** CRISPR/Cas9 functional genomics studies relevant to psychotropic drug response and pharmacogenetics.

Gene(s) edited	Cell model	CRISPR method	Drug(s) tested	Key finding	References
*SLC6A4*, *HTR2A*, pooled library (>100 genes)	iPSC-derived serotonergic neurons	CRISPRi/a (dCas9-KRAB/dCas9-VP64)	Fluoxetine (SSRI)	Modulation of *SLC6A4* expression altered serotonin reuptake capacity and cellular response to SSRIs; identified gene networks modulating antidepressant sensitivity	Tian et al. (2019)
*SETD1A*, *NRXN1*, *DISC1*	iPSC-derived cortical neurons	CRISPR knockout (Cas9)	Clozapine (atypical antipsychotic)	Risk variants in schizophrenia-associated genes altered synaptogenesis, neuronal excitability, and cellular response to clozapine	Kurishev et al. (2022)
Pooled library (>2,500 enhancers)	iPSC-derived excitatory neurons	CRISPRi (dCas9-KRAB)	SSRIs, antipsychotics (multiple)	Systematic perturbation identified gene–drug interaction networks and regulatory elements influencing psychotropic response	Norman et al. (2019)
*BDNF* (Val66Met variant)	iPSC-derived neurons	CRISPR knock-in (homology-directed repair)	Multiple antidepressants (SSRIs, SNRIs)	Val66Met variant affected neurotrophin signaling and altered cellular response to antidepressants	Shi et al. (2012)
*CYP2D6*, *CYP2C19* (functional alleles)	iPSC-derived hepatocyte-like cells and neurons	CRISPR knock-in/ knock-out	Risperidone, aripiprazol, fluoxetine	Introduced poor metabolizer (CYP2D6*4) and ultra-rapid (*CYP2D6* duplication) alleles; quantified enzymatic activity and drug clearance differences	Gutiérrez-Rodríguez et al. (2023)
*COMT*, *DRD2*, *SLC6A4*	iPSC-derived dopaminergic and serotonergic neurons	CRISPRa (dCas9-VP64)	Haloperidol, olanzapine	Upregulation of *COMT* and *DRD2* altered dopamine signaling and antipsychotic sensitivity	Tian et al. (2019)
*HTR2A* (rs6313, rs6311)	HEK293 cells, iPSC-derived neurons	CRISPR knock-in (HDR)	Clozapine, risperidone	Functional variants in *HTR2A* promoter altered receptor expression and antipsychotic response	Dominguez et al. (2016)

Future perspectives involve the integration of genome editing, transcriptomics, and high-resolution neuronal models to map pharmacogenetically relevant pathways with greater accuracy. When applied systematically, CRISPR-based functional genomics provides the experimental evidence necessary to strengthen genotype-to-phenotype predictions, particularly in under-represented or highly admixed populations where the clinical relevance of many variants remains uncertain. By shifting pharmacogenetics from associative to mechanistic evidence, these tools contribute to the development of more reliable and equitable approaches to personalized psychotropic treatment.

## 4 Clinically relevant pharmacogenetic variants, CRISPR functional genomics, and admixture-aware psychiatry in Brazil

Extensive evidence demonstrates that polymorphisms in pharmacogenes such as *CYP2D6* and *CYP2C19* substantially influence the metabolism of widely prescribed antidepressants and antipsychotics. These cytochrome P450 enzymes determine plasma concentrations of medications including fluoxetine, sertraline, and risperidone, and thereby contribute to both therapeutic success and the risk of adverse effects (van Westrhenen et al., 2020). Variants that confer ultra-rapid metabolism often reduce drug exposure and compromise efficacy, whereas variants associated with poor metabolism increase the likelihood of toxicity. In recognition of this, the Clinical Pharmacogenetics Implementation Consortium (CPIC) and the Dutch Pharmacogenetics Working Group (DPWG) recommend genotype-informed dosing for several psychotropic agents. Nonetheless, the uptake of these guidelines remains limited due to barriers including standardization, clinician training, and the absence of integrated decision-support systems in psychiatric practice.

Beyond pharmacokinetics, pharmacodynamic genes also shape patient response to psychotropic medications. Variants in *SLC6A4* and *HTR2A*, for example, have been associated with differential responses to selective serotonin reuptake inhibitors (SSRIs). The 5-HTTLPR polymorphism in *SLC6A4* remains one of the most studied predictors of SSRI efficacy yet meta-analytic results demonstrate considerable heterogeneity across studies and populations (Porcelli et al., 2011). This inconsistency reflects the multifactorial nature of psychotropic response, which integrates genetic background, environmental influences, comorbidities, polypharmacy, and treatment adherence. Genome-wide association studies (GWAS) continue to identify additional variants associated with antidepressant or antipsychotic response, but functional validation remains scarce, particularly in non-European populations (Ahmad et al., 2024).

To address these limitations, polygenic and multimodal predictive frameworks have been proposed. van Westrhenen et al. (2020) argue that combining genetic, clinical, and environmental data through machine-learning approaches may improve the accuracy of treatment predictions relative to traditional single-gene models. Complementing this, patient-derived induced pluripotent stem cell (iPSC) models increasingly serve as experimental platforms for assessing the biological impact of pharmacogenetic variants.

Within this context, CRISPR/Cas9 technology has emerged as a critical tool for establishing causal relationships between genotype and pharmacological response. As discussed by Gutiérrez-Rodríguez et al. (2023), CRISPR enables the precise introduction, deletion, or modification of alleles in human cellular systems, including iPSC-derived neurons. By generating isogenic lines differing only at a specific locus, investigators can directly assess how candidate variants influence gene expression, intracellular signaling, synaptic properties, or drug sensitivity. This mechanistic insight complements clinical association studies and helps clarify whether particular alleles meaningfully alter antidepressant or antipsychotic response.

Non-mutagenic CRISPR modalities—CRISPR interference (CRISPRi) and CRISPR activation (CRISPRa)—further expand the capacity to examine regulatory variants that modulate transcription rather than protein structure. These approaches allow reversible, dose-dependent control of gene expression, making them suitable for interrogating variants in genes such as SLC6A4 and COMT that influence psychotropic response through altered expression levels (Tian et al., 2019) By adjusting gene dosage within controlled neuronal environments, researchers can quantify downstream effects on drug efficacy, toxicity, and signaling pathways relevant to SSRIs, SNRIs, and antipsychotics.

High-throughput CRISPR screening platforms extend this analysis to polygenic architectures. Pooled CRISPRi/a libraries allow the simultaneous perturbation of hundreds of genes, followed by phenotypic readouts such as transcriptomics, morphology, electrophysiology, or drug-response profiling (Norman et al., 2019). Exposing these perturbed neuronal libraries to SSRIs or atypical antipsychotics enables systematic identification of gene–drug interactions, resistance mechanisms, and cellular pathways that shape psychotropic response (Tian et al., 2019) These experiments offer a scalable strategy to functionally annotate GWAS findings and refine which loci exert true biological effects on pharmacodynamics. CRISPR-based functional genomics has also been applied to psychiatric risk genes implicated in neurodevelopmental pathways. Studies such as Kurishev et al. (2022) demonstrate that editing variants in genes including SETD1A and NRXN1 alters neuronal morphology, synaptogenesis, and excitability—traits that may indirectly influence antipsychotic response. While these studies primarily address disease pathophysiology rather than drug metabolism *per se*, their methodological advances—particularly isogenic editing in neuronal lineages—are directly transferable to pharmacogenetic research.

CRISPR-edited patient-derived neuronal models therefore offer a translational bridge toward individualized psychopharmacology. By recreating patient-specific variant combinations *in vitro* and exposing these models to psychotropic drugs, it becomes possible to anticipate differential cellular responses prior to clinical treatment. Such strategies remain experimental but highlight a future in which CRISPR-based functional evidence informs more accurate and equitable medication selection. Collectively, these approaches illustrate how CRISPR technologies—applied not as clinical therapies but as mechanistic tools—can clarify the functional impact of pharmacogenetic variants, support the refinement of predictive models, and ultimately enhance the precision of antidepressant and antipsychotic prescribing.

The large-scale implementation of pharmacogenetics in psychiatry, however, continues to face substantial challenges, particularly in countries characterized by extensive genetic heterogeneity. In Brazil, centuries of admixture involving European, African, and Indigenous populations have produced one of the most complex genomic landscapes globally. This diversity complicates the interpretation of pharmacogenetic variants and limits the direct applicability of international guidelines derived from more homogeneous populations (Suarez-Kurtz et al., 2012). Such heterogeneity is especially consequential for psychotropic pharmacogenetics, in which allelic variation in CYP2D6, CYP2C19, SLC6A4, and DRD2 plays a decisive role in shaping antidepressant and antipsychotic response. Integrating CRISPR functional genomics with admixture-aware psychiatry offers a mechanistic pathway to overcome these limitations by enabling direct experimental testing of population-specific and low-frequency variants within controlled cellular models. Functional characterization of these alleles, particularly those enriched in admixed Brazilian genomes, can refine phenotype prediction and provide evidence better aligned with the country’s complex genetic architecture (Matos et al., 2020; Rajarajan et al., 2020).

In North America and Europe, genetics is increasingly incorporated into clinical workflows through standardized guidelines, notably those produced by CPIC and DPWG. These frameworks recommend genotype-informed dosing adjustments and highlight gene–drug pairs with demonstrated clinical relevance (Thottunkal et al., 2025). However, applying these guidelines directly to Brazil may lead to misclassification of metabolizer phenotypes due to population-specific allele frequencies and ancestry-dependent haplotype structures (Fernandes et al., 2021). Without local calibration and functional validation, clinical decision-making may inadvertently perpetuate inequities. CRISPR-enabled functional assays can address this gap by experimentally determining whether Brazilian-specific variants produce metabolic profiles equivalent to known alleles—such as distinguishing whether a rare CYP2D6 haplotype behaves like *4 (non-functional) or *41 (reduced function)—thereby informing more accurate, ancestry-aware clinical classification (Bertholim-Nasciben et al., 2023).

Structural and systemic barriers also limit the integration of pharmacogenetics into Brazilian psychiatric practice. These include restricted access to genetic testing, insufficient continuing medical education for healthcare professionals, and the absence of consolidated public policies within the Brazilian Unified Health System (SUS). Although initiatives such as the National Pharmacogenetics Network (Refargen) represent meaningful progress, they are constrained by fragmented infrastructure, limited funding, and a lack of coordination between molecular laboratories, clinical services, and public health authorities (Suarez-Kurtz et al., 2014). These gaps are particularly problematic when considering drugs with narrow therapeutic windows or pronounced interindividual variability, where pharmacogenetics could provide substantial clinical benefit.

Despite these barriers, the genetic diversity of Brazil also offers unique opportunities. Admixed populations provide a natural model for studying the functional effects of variants that are rare in European cohorts yet common in Brazilian genomic backgrounds. This diversity can inform the development of more inclusive dosing guidelines, refine genotype–phenotype predictions, and support ancestry-aware clinical algorithms for psychotropic medications. When paired with CRISPR-based functional genomics, this diversity becomes even more valuable, enabling systematic validation of alleles within isogenic systems and reinforcing precision-oriented frameworks grounded in local genetic realities. As psychopharmacology increasingly moves toward precision-oriented frameworks, incorporating admixed genomes into evidence generation becomes essential for both scientific rigor and ethical implementation.

In summary, the integration of pharmacogenetics into Brazilian psychiatry demands more than scientific advances alone. It requires sustained investment in genomic infrastructure, coordinated public policies, equitable access to testing, and the systematic inclusion of genetically diverse populations in research. A deeper understanding of variants in genes such as CYP2D6, CYP2C19, SLC6A4, and DRD2—combined with CRISPR-based functional genomics and explicit consideration of Brazil’s admixture—will be indispensable for improving therapeutic outcomes and advancing a more precise, effective, and socially contextualized model of psychiatric care.

## 5 Genetic admixture in Brazil and implications for pharmacogenetics

The clinical application of pharmacogenetics in highly heterogeneous populations, such as Brazil’s, encounters significant challenges. This genetic complexity arises from historical admixture involving European, African, and Indigenous populations, resulting in one of the most diverse genetic landscapes globally (Suarez-Kurtz et al., 2012). Such diversity directly impacts the interpretation of pharmacogenetic tests and the accuracy of predicting individual responses to psychotropic medications, including antidepressants and antipsychotics.

Population-based studies in Brazil, such as those conducted by the National Pharmacogenetics Network (Refargen), have identified a broad spectrum of functional variants in genes like *CYP2D6*, *CYP2C19*, *CYP3A5*, and *NAT2* (Table 3). These variants significantly influence the metabolism of psychotropic drugs. For instance, the CYP3A5*3 allele, associated with reduced enzymatic activity, exhibits variable frequencies depending on ancestry proportions. Higher European ancestry correlates with a greater prevalence of this allele, whereas African ancestry is associated with lower frequencies (Bonifaz-Peña et al., 2014; Suarez-Kurtz et al., 2014).

**TABLE 3:** Regional variation in pharmacogenetic allele frequencies across Brazil, reflecting ancestral admixture gradients.

Gene/ Variant	Ancestral origin	European-ancestry brazilians (%)	African-ancestry brazilians (%)	Indigenous-ancestry brazilians (%)	Predominant regional distribution	Key references
CYP2D6*4 (non-functional)	European	10–12	2–4	<1	South (rio grande do Sul, Santa Catarina)	Suarez-Kurtz et al. (2012), Suarez-Kurtz et al. (2014)
CYP2D6*17 (reduced activity)	African	<1	8–12	<1	Northeast (Bahia, Pernambuco)	Bonifaz-Peña et al. (2014), Suarez-Kurtz, (2010)
CYP2D6*5 (gene deletion)	European/African	4–6	3–5	<1	Southeast (minas gerais, São Paulo)	Suarez-Kurtz et al. (2012)
CYP2C19*2 (loss-of-function)	European/Asian	12–16	6–10	4–6	Southeast (São Paulo, rio de janeiro)	Suarez-Kurtz et al. (2012), Fernandes et al. (2021)
CYP2C19*3 (loss-of-function)	Asian	<1	<1	1–3	North (Amazonas, Pará)	Fernandes et al. (2021)
CYP2C19*17 (gain-of-function)	European	18–24	10–14	8–12	South and central-west	Bonifaz-Peña et al. (2014), Suarez-Kurtz et al. (2014)
CYP3A5*3 (reduced activity)	European	65–75	25–35	15–25	Functional *1 allele more common in northeast and north	Bonifaz-Peña et al. (2014), Suarez-Kurtz, (2010)
NAT2*5 (slow acetylator)	European	40–48	28–35	22–28	South and Southeast	Suarez-Kurtz et al. (2012)
NAT2*7 (slow acetylator)	African	3–6	10–14	3–5	Northeast	Bonifaz-Peña et al. (2014)
SLCO1B1*5 (reduced transporter)	European	12–16	6–10	4–8	South	Suarez-Kurtz et al. (2014)
SLC6A4 5-HTTLPR “s” allele	European/African	38–44	32–38	28–34	Slightly higher in South and Southeast	Crawford et al. (2013), Bonifaz-Peña et al. (2014)

This genetic diversity complicates the accurate classification of metabolic profiles, leading to uncertainty in predicting drug efficacy and adverse effects. Sociocultural criteria traditionally used to estimate genetic ancestry often lack precision, underscoring the importance of genomic ancestry analysis for predicting pharmacogenetic variant distributions in admixed populations (Suarez-Kurtz et al., 2012).

Brazil’s admixture pattern is notably more complex than that of other Latin American countries like Mexico, Peru, and Chile. While these countries exhibit predominant Indigenous ancestry in certain regions, Brazil displays a more balanced representation of European, African, and Indigenous ancestries, with significant regional variation (Bonifaz-Peña et al., 2014). This complexity further challenges the direct application of international guidelines without local adaptation.

Polygenic risk scores (PRS) and combinatorial pharmacogenetic algorithms face additional limitations in admixed populations. As (Martin et al., 2019) demonstrated, PRS trained on European-ancestry cohorts exhibit significantly reduced predictive accuracy in non-European groups due to allele frequency divergence, haplotype structure differences, and incomplete LD transfer. In Brazil, this ancestry-dependent performance decay may obscure gene-drug interactions beyond core pharmacogenes (e.g., *EPHX1*, *ABCB1*), reinforcing the need for ancestry-aware polygenic models.

Latin American populations remain underrepresented in global genomic databases, which are foundational to many drug response prediction algorithms. This underrepresentation compromises the accuracy of pharmacogenetic tools used in Brazilian clinical practice, as many common variants in admixed populations are inadequately documented (Fernandes et al., 2021).

From a clinical perspective, pharmacogenetics holds the potential to reduce therapeutic failure rates, minimize adverse effects, and enhance medication adherence. However, these benefits can only be equitably realized if Brazil’s genetic diversity is fully integrated into research, databases, and therapeutic guidelines.

In conclusion, the genetic admixture and variability in Brazil pose significant challenges to the implementation of pharmacogenetics. Yet, they also present a unique opportunity for developing innovative and inclusive solutions. Investment in national scientific research, genomic infrastructure, and specialized professional training will be crucial to ensuring safer, more effective treatments for all Brazilians.

## 6 Foundations of pharmacogenetics in psychotropic medications

Pharmacogenetics seeks to determine how interindividual genetic variation influences drug response, a question of particular relevance in psychiatry, where antidepressants and antipsychotics demonstrate wide variability in efficacy and adverse event profiles (Zhou et al., 2009; Kimura et al., 2022). Much of this variability arises from polymorphisms in genes involved in drug metabolism, neurotransmitter transport, and receptor signaling. Cytochrome P450 enzymes, especially CYP2D6 and CYP2C19, remain central to this framework, as they mediate the metabolism of tricyclic antidepressants, selective serotonin reuptake inhibitors (SSRIs), and multiple antipsychotics (Zanger and Schwab, 2013).

**FIGURE 2:**
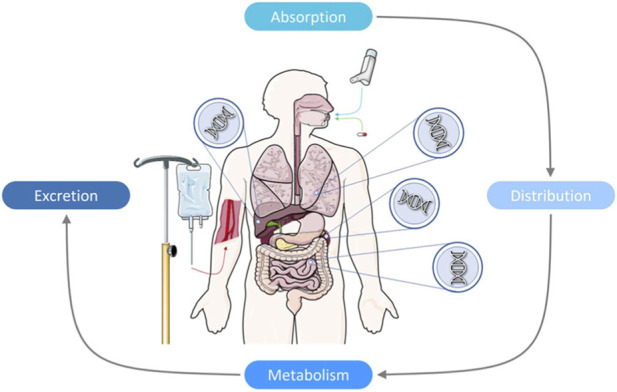
Illustration of the effects of genetic polymorphisms on the pharmacokinetic stages of psychotropic drugs, including absorption, hepatic metabolism, transport, and elimination. The gene–drug interaction directly influences therapeutic efficacy, highlighting the importance of the genetic profile in treatment response (Source: ZHOU, S. F. Polymorphism of human cytochrome P450 2D6 and its clinical significance. Drug Metabolism Reviews, vol. 41, no. 1, pp. 89–295, 2009).

Allelic variation in these genes stratifies individuals into distinct metabolizer phenotypes with direct consequences for plasma drug concentrations and clinical outcomes. For CYP2D6, current guidelines typically distinguish four categories—poor, intermediate, normal/extensive, and ultra-rapid metabolizers—whereas for CYP2C19 five phenotypes are recognized: poor, intermediate, normal/extensive, rapid, and ultra-rapid metabolizers (Hicks et al., 2017; Bousman et al., 2023b). Carriers of duplicated functional *CYP2D6* alleles may clear medications more rapidly, risking subtherapeutic exposure, whereas individuals with nonfunctional alleles may accumulate the drug, increasing susceptibility to toxicity (Chan et al., 2019). These dynamics underscore the clinical relevance of genotyping for dose selection and drug choice. As depicted in Figure 2, polymorphisms can modulate multiple pharmacokinetic phases—including absorption, hepatic metabolism, transport, and elimination—ultimately shaping interindividual treatment response.

In addition to metabolic pathways, variants in genes involved in serotonergic and dopaminergic signaling have been extensively studied. Polymorphisms in the *SLC6A4* promoter, particularly the 5-HTTLPR locus, have been associated with differential SSRI response, with the short (s) allele linked to reduced efficacy and increased risk of adverse effects in some cohorts (Crawford et al., 2013). For antipsychotics, variants in *DRD2*, encoding the dopamine D2 receptor, and *COMT*, involved in dopamine degradation, have been associated with variability in symptom improvement and extrapyramidal side effects (Rampino et al., 2019; Taheri et al., 2023; Xing et al., 2007). Collectively, these associations highlight the complexity of psychotropic drug response, which emerges from the interaction of multiple genes and environmental factors.

Operationally, an ancestry-aware CRISPR-enabled pipeline for Brazil (figure) would proceed in four steps: (1) large-scale sequencing of psychiatric cohorts across Brazilian macro-regions, with explicit estimation of local ancestry and identification of population-specific pharmacogenetic haplotypes (particularly in CYP2D6, CYP2C19, CYP3A5, SLC6A4, DRD2 and COMT); (2) prioritization of variants with uncertain function but high frequency or effect size for CRISPR-based knock-in/knock-out or CRISPRi/a experiments in iPSC-derived hepatocytes and neurons; (3) quantification of variant-specific effects on enzyme activity, drug clearance, receptor signaling and cellular drug response using standardized assays; and (4) integration of these functional effect sizes into revised genotype-to-phenotype translation tables and dosing recommendations tailored to Brazilian haplotype structures and ancestry gradients. Such a pipeline would provide a transparent route from variant discovery to clinically actionable, locally calibrated prescribing guidelines

**FIGURE 3:**
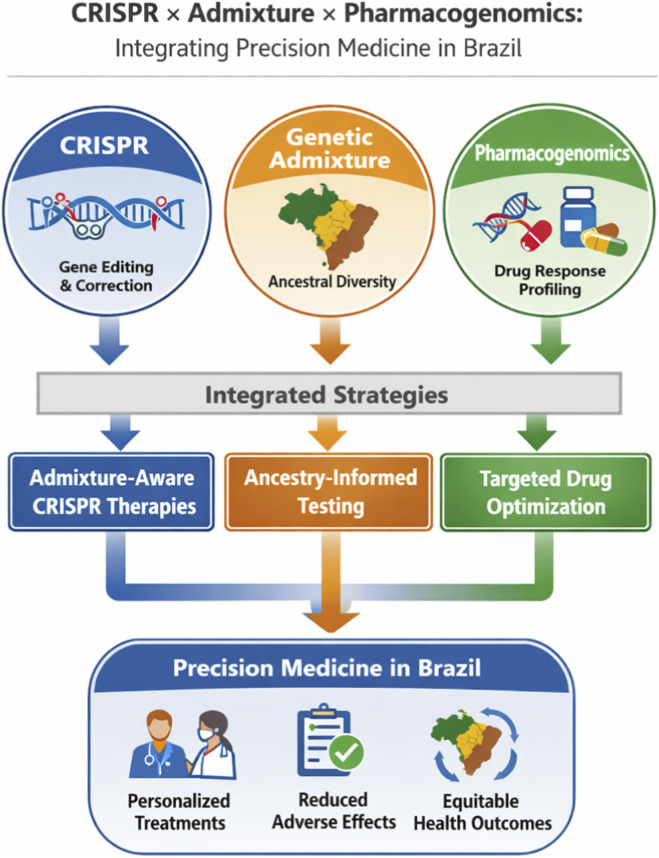
Integrating CRISPR/Cas9 functional genomics with admixture-aware pharmacogenetics in Brazil. Schematic workflow illustrating how CRISPR/Cas9 technologies can be integrated into pharmacogenetic research to address challenges posed by genetic admixture in Brazilian populations. Brazilian ancestry gradient: geographic distribution of European (blue), African (red), and Indigenous (green) ancestral components across macro-regions (South, Southeast, Central-West, Northeast, North). Variant discovery and characterization: sequencing of admixed cohorts identifies population-specific and rare alleles in key pharmacogenes (*CYP2D6*, *CYP2C19*, *SLC6A4*, *HTR2A*, *DRD2*, *COMT*). CRISPR functional validation: genome editing (knock-in, knock-out, CRISPRi/a) introduces variants into isogenic iPSC-derived neuronal and hepatic models. Phenotypic readouts: measurement of drug metabolism (LC-MS/MS), neurotransmitter signaling (patch-clamp, calcium imaging), and cellular drug response (viability, gene expression). Ancestry-informed guidelines: integration of functional data with population-specific allele frequencies and polygenic prediction to refine CPIC/DPWG recommendations for Brazilian psychiatric patients. This integrated approach strengthens causal inference, improves predictive accuracy, and supports equitable, personalized prescribing across genetically diverse populations.

In summary, the foundational principles of pharmacogenetics provide a mechanistic basis for refining psychotropic prescribing by incorporating genotypic information on key genes such as *CYP2D6*, *CYP2C19*, *SLC6A4*, and *DRD2* (Figure 3). As psychiatric treatment increasingly moves toward precision medicine, these principles offer an essential pathway for improving therapeutic efficacy and minimizing adverse effects.

## 7 Conclusion

Pharmacogenetics offers a robust framework to improve the safety and effectiveness of antidepressants and antipsychotics, but its clinical impact is highly context-dependent. In Brazil, the extensive admixture among European, African, and Indigenous populations generates a genomic architecture that diverges from the largely European cohorts on which most international guidelines are based, limiting the direct applicability of ancestry-agnostic prescribing frameworks and increasing the risk of phenotype misclassification. This scenario underscores three central messages from this review: first, that ancestry-dependent variation in pharmacogenes such as CYP2D6, CYP2C19, CYP3A5, SLC6A4, and DRD2 has major implications for psychotropic prescribing; second, that CRISPR-based functional genomics can provide the mechanistic evidence needed to resolve uncertainties in variant classification, particularly for alleles enriched in admixed Brazilian genomes; and third, that equitable implementation will require explicit integration of admixture-aware data into clinical decision-making.

By combining CRISPR-mediated knock-in, knock-out, and CRISPRi/a approaches with iPSC-derived hepatocyte and neuronal models, it becomes possible to experimentally define the metabolic, regulatory, and pharmacodynamic consequences of population-specific variants. These functional datasets can refine metabolizer phenotype prediction, clarify whether rare Brazilian haplotypes behave as non-functional, reduced-function, or gain-of-function alleles, and support the development of dosing guidelines calibrated to local haplotype structures and ancestry gradients. In doing so, CRISPR functional genomics transforms pharmacogenetics from a largely associative discipline into a mechanistic, experimentally anchored framework that is better suited to the complexity of highly admixed populations.

Building a truly admixture-aware, CRISPR-informed psychopharmacology in Brazil will depend on a small set of strategic priorities. First, research efforts should focus on systematically mapping and functionally characterizing pharmacogenetic variants in diverse Brazilian cohorts, integrating genomic, transcriptomic, and cellular phenotyping data. Second, national and regional guidelines should be revised to incorporate both ancestry-stratified allele frequency information and CRISPR-derived functional annotations, rather than relying on direct extrapolation from predominantly European datasets. Third, policy initiatives should prioritize equitable access to pharmacogenomic testing and decision-support tools, alongside targeted education for psychiatrists, primary-care providers, and pharmacists on ancestry- and equity-conscious prescribing. If aligned, these scientific and policy agendas can position Brazil as a model for integrating functional genomics and population diversity into precision psychiatry, advancing psychotropic treatment that is not only more precise, but also more locally calibrated and socially just.
